# The Story of the Insane from Year to Year

**Published:** 1895-06-01

**Authors:** 


### THE STORY OF THE INSANE FROM YEAR TO YEAR.

(7th Series.)

### ST. LUKE'S HOSPITAL FOR LUNATICS.

Having been founded in 1751 this is one of the oldest lunatic asylums in the country. During the year 1893 the numbers rose from 186 to 191. There were 67 admissions, 24 recoveries, and 10 deaths. The recoveries stand at 37·50 per cent. on the admissions. A convalescent establishment has been opened at Ramsgate and Dr. Mickley speaks very highly of the advantages accruing from it and looks upon it as a valuable acquisition to the hospital. Many improvements have been carried out during the year, and the committee seem determined to maintain the efficiency of the asylum. The attendants and nurses have been put into uniforms, and it is intended to improve the accommodations for the female staff which is not satisfactory at present. Of the year's admissions 42 were from the metropolis, 24 from the provinces, and 1 from Germany. All sorts and conditions of men and women were represented. We notice publicans, commission agents, journalists, saddlers, clerks, storekeepers, clergymen, watchmakers, engineers, dressmakers, barmaids, governesses, nurses, music-teachers, and tie-makers. The asylum is well-manned medically, for, in addition to the medical superintendent, there are one assistant medical officer and two clinical clerks.

